# Interferon-stimulated gene TDRD7 interacts with AMPK and inhibits its activation to suppress viral replication and pathogenesis

**DOI:** 10.1128/mbio.00611-23

**Published:** 2023-09-15

**Authors:** Sukanya Chakravarty, Gayatri Subramanian, Sonam Popli, Manoj Veleeparambil, Shumin Fan, Ritu Chakravarti, Saurabh Chattopadhyay

**Affiliations:** 1 Department of Medical Microbiology and Immunology, University of Toledo College of Medicine and Life Sciences, Toledo, Ohio, USA; 2 Department of Physiology and Pharmacology, University of Toledo College of Medicine and Life Sciences, Toledo, Ohio, USA; The University of North Carolina at Chapel Hill, Chapel Hill, North Carolina, USA

**Keywords:** Innate immunity, interferon, ISGs, TDRD7, AMPK, autophagy, paramyxovirus, herpesvirus

## Abstract

Many viruses activate cellular autophagy in infected cells to facilitate their replication. Recently, we identified an interferon (IFN)-stimulated gene (ISG) Tudor domain containing 7 (TDRD7), which inhibits viral replication by blocking autophagy pathway. Here, we present a molecular mechanism for TDRD7 action and its relative contribution to protection against viral pathogenesis. TDRD7 inhibited the activation of adenosine monophosphate (AMP)-activated protein kinase (AMPK), a kinase required for initiating autophagy. Mechanistically, TDRD7 interacted directly with AMPK in the cytosolic compartment. Domain-mapping analyses revealed C-terminal Tudor domain of TDRD7 interacted with auto-inhibitory domain of AMPK. Deletion of Tudor domains abolished anti-AMPK and antiviral activities of TDRD7. We evaluated physiological relevance of TDRD7 function against viral replication using newly engineered TDRD7 knockout mice and the derived primary cells. TDRD7 knockout primary cells displayed increased AMPK activation, which led to a higher viral load. Subsequently, TDRD7 knockout mice showed enhanced susceptibility upon intranasal Sendai virus infection. Therefore, our study revealed a new antiviral function of IFN, mediated by TDRD7-AMPK, inhibiting viral replication and pathogenesis.

**IMPORTANCE:**

Virus infection triggers induction of interferon (IFN)-stimulated genes (ISGs), which ironically inhibit viruses themselves. We identified Tudor domain-containing 7 (TDRD7) as a novel antiviral ISG, which inhibits viral replication by interfering with autophagy pathway. Here, we present a molecular basis for autophagy inhibitory function of TDRD7. TDRD7 interacted with adenosine monophosphate (AMP)-activated protein kinase (AMPK), the kinase that initiates autophagy, to inhibit its activation. We identified domains required for the interaction; deleting AMPK-interacting domain blocked antiAMPK and antiviral activities of TDRD7. We used primary cells and mice to evaluate the TDRD7-AMPK antiviral pathway. TDRD7-deficient primary mouse cells exhibited enhanced AMPK activation and viral replication. Finally, TDRD7 knockout mice showed increased susceptibility to respiratory virus infection. Therefore, our study revealed a new antiviral pathway of IFN and its contribution to host response. Our results have therapeutic potential; a TDRD7-derived peptide may be an effective AMPK inhibitor with application as antiviral agent.

## INTRODUCTION

Virus infection causes rapid activation of pattern recognition receptors (PRRs), e.g., TLR, RLR, cGAS, and STING, which sense viral nucleic acids and trigger intracellular signaling pathways (1 – 3). PRRs activate IRF3 and NF-κB, transcription factors inducing the interferon (IFN) genes, e.g., IFNβ (4 – 6). IFNs get secreted and act on infected or yet-uninfected neighboring cells and induce expression of IFN-stimulated genes (ISGs). Many ISGs function as viral restriction factors by directly interfering with specific stages of viral replication. Some antiviral ISGs regulate cellular proteins, which viruses use for their benefit. It has become increasingly clear that ISGs act virus specifically to exhibit antiviral effects. High throughput genetic screens are carried out to identify virus-specific ISGs (7, 8). We performed a high throughput screen of human ISGs and isolated a novel viral restriction factor, Tudor domain containing 7 (TDRD7) (9). TDRD7 inhibits replication of SeV, RSV, HPIV3, and herpes simplex virus 1 (HSV-1) in human and mouse cells (9, 10). Antiviral function of TDRD7 depends on its ability to inhibit virus-activated autophagy, a cellular catabolic pathway. We showed that TDRD7 interferes with initiation step of autophagy pathway, induced by viruses.

Autophagy is initiated by activation of adenosine monophosphate (AMP)-activated protein kinase (AMPK), a Ser/Thr kinase. Low cellular ATP levels lead to increased AMP, which activates AMPK triggering, in addition to autophagy, an array of downstream signaling pathways, e.g., mTOR, SIRT1, and GLUT (11 – 13). Many viruses use activated AMPK during their replication (14 – 16). Human cytomegalovirus (HCMV) activates AMPK to promote viral replication; however, the exact pro-HCMV mechanism is unclear (17 – 19). A recent study suggests that HCMV infection induces AMPKα2 to activate glycolytic pathway to support viral replication (18). Kaposi's sarcoma-associated herpesvirus (KSHV) regulates AMPK activity using viral K1 protein to enhance cell survival for promoting viral replication (20). On the other hand, hepatitis C virus (HCV) replication is inhibited by AMPK (21). How AMPK is involved in virus replication is unclear, and the multifunctional nature of AMPK suggests that no single mechanism may be responsible for its virus-regulating activity. We showed paramyxo/pneumo viruses, e.g., Sendai virus (SeV), respiratory syncytial virus (RSV), and human parainfluenza virus 3 (HPIV3), utilize AMPK-dependent autophagy pathway for viral replication (9). In contrast, HSV-1 activates AMPK and uses an autophagy-independent pathway of AMPK for viral replication (10). In addition to directly influencing viral life cycle, AMPK regulates innate immune response to virus infection. Cytosolic DNA-sensor STING functions are regulated by AMPK (22, 23). AMPK also directly phosphorylates JAK1 to inhibit pro-inflammatory signaling pathway (24).

AMPK is a heterotrimeric complex, consisting of catalytic (α) and regulatory (β and γ) subunits and is activated during energy-deprived cellular conditions (11, 13). AMPK is activated in cells by both canonical and noncanonical pathways. In canonical pathway, AMPK is activated by increase in cellular AMP or Ca^2+^ levels, which are associated with nutrient deprivation, cellular stress, and viral infection. In addition to direct activation, AMPK can be activated by pharmaceuticals and chemical agents. Metformin, an antidiabetic compound currently used on patients, activates AMPK by increasing cellular AMP levels. AMPK can be activated noncanonically by DNA damage or reactive oxygen species (ROS), which are not associated with increase in AMP or Ca^2+^ levels. In addition, A-769662, identified using a high throughput screen, binds AMPK directly to activate it. AICAR, a chemical agent, upon cellular uptake, produces an AMP analog that activates AMPK. Among kinases known to phosphorylate AMPK, liver kinase B1 (LKB1) is the most well-studied. LKB1 gets recruited to the AMPK-activating complex in presence of increased cellular AMP (12). In presence of high AMP, LKB1 gets recruited to lysosome membrane, a platform for AMPK-activating complex. Activated by LKB1, AMPK dissociates from lysosome membrane to phosphorylate downstream targets, e.g., mTOR (12). Activated AMPK phosphorylates ULK1, the kinase critical for initiation of autophagy. In the current study, we investigated how TDRD7 inhibits activation of AMPK. TDRD7 interacted with AMPK, and the interaction was required for antiAMPK and, subsequently, antiviral function of TDRD7. We also evaluated the role of TDRD7 in suppressing viral pathogenesis using newly engineered TDRD7 conditional knockout (KO) mice. Our results from *ex vivo* and *in vivo* studies demonstrated the role of TDRD7/AMPK interaction in protection against viral infection.

## RESULTS

### IFN-stimulated gene TDRD7 interacts with AMPK and inhibits its activation

**Fig 1:**
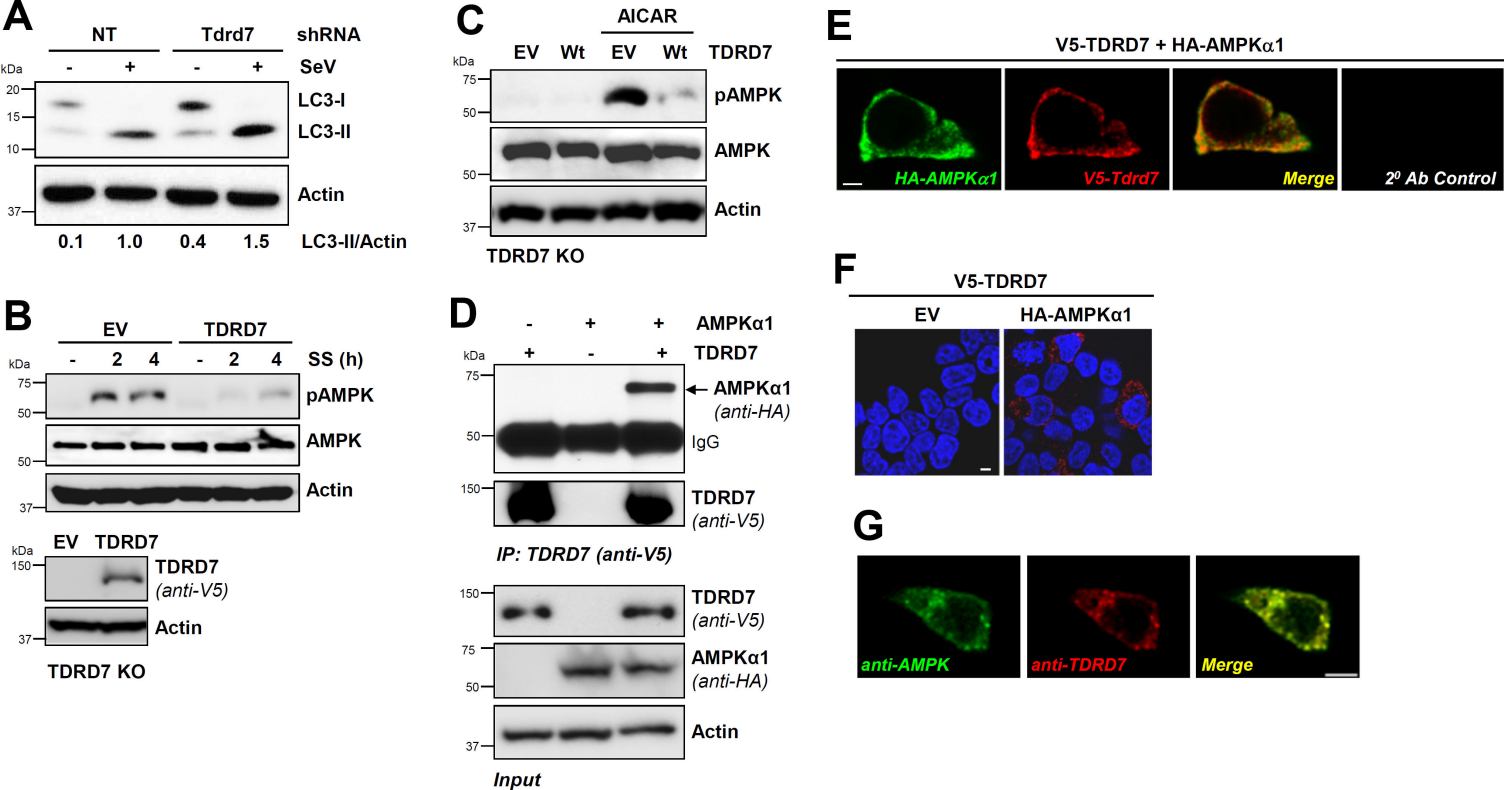
TDRD7 interacts with AMPK and inhibits its activation by various stimuli. (**A**) L929 cells expressing Tdrd7-specific shRNA were infected with SeV, and LC3-II was analyzed by immunoblot at 8 hours post-infection (hpi). (**B**) TDRD7 knockout (KO) cells, ectopically expressing V5-TDRD7, were serum starved and analyzed for pAMPK by immunoblot. (**C**) TDRD7 KO cells, ectopically expressing V5-TDRD7, were treated with AICAR and analyzed for pAMPK by immunoblot 2 h post-treatment. (**D**) HEK293T cells were co-transfected with V5-TDRD7 and HA-AMPKα1 plasmids. TDRD7 was immunoprecipitated from the cell lysates 24 h post-transfection, and the immunoprecipitates were analyzed for AMPK by immunoblot. (**E**) HEK293T cells were co-transfected with V5-TDRD7 and HA-AMPKα1 plasmids. TDRD7 and AMPK were immuno-stained and analyzed by confocal microscopy. (**F**) HEK293T cells were co-transfected with V5-TDRD7 and HA-AMPKα1 plasmids, and TDRD7:AMPK interaction was analyzed by duolink assay; the red dots indicate the duolink signals. (**G**) HT1080 cells were immuno-stained using anti-TDRD7 and antiAMPK antibodies and analyzed by confocal microscopy. NT, nontargeting, EV, empty vector; scale bar, 5 µm. The results are representative of at least three experiments.

In recent studies, we reported TDRD7 as a new viral restriction factor, which inhibits cellular autophagy pathway to suppress viral replication (9, 10). Tdrd7 knockdown mouse cells, as expected, displayed increased LC3-II, a marker of autophagy, upon SeV infection compared to control cells (Fig. 1A, lanes 2 and 4). We noted increased LC3-II levels in Tdrd7-knockdown uninfected cells (Fig. 1A, lanes 1 and 3), indicating Tdrd7 also inhibited basal autophagy. We previously showed TDRD7 blocks autophagy by interfering with the autophagy-initiating kinase AMPK; however, the mechanism is unknown. We used serum starvation (SS) and AICAR, two known activators of AMPK, for investigating TDRD7-mediated AMPK inhibition. SS-induced activation of AMPK, analyzed by its phosphorylation on Thr^172^ (pAMPK) in TDRD7 knockout cells, was inhibited by restoration of TDRD7 expression (Fig. 1B). TDRD7 expression also inhibited pAMPK, induced by AICAR, a pharmaceutical activator of AMPK (Fig. 1C). To investigate the molecular basis of antiAMPK activity of TDRD7, we inquired whether TDRD7 and AMPK formed a complex. We used ectopic co-expression of epitope-tagged TDRD7 and AMPK in HEK293T cells, followed by co-immunoprecipitation (co-IP). Our results indicate AMPK co-immunoprecipitated with TDRD7 (Fig. 1D). We validated co-IP results using confocal microscopy, which showed robust co-localization of AMPK and TDRD7 proteins (Fig. 1E). We confirmed these results by proximity ligation assay, which detects closely localized proteins (Fig. 1F). Similar to ectopically expressed proteins, endogenous TDRD7 and AMPK proteins also co-localized, as analyzed by confocal microscopy (Fig. 1G). Together, TDRD7-mediated inhibition of AMPK was accompanied by their interaction.

### TDRD7 interacts with regulatory domain of AMPK via its C-terminal Tudor domain

**Fig 2:**
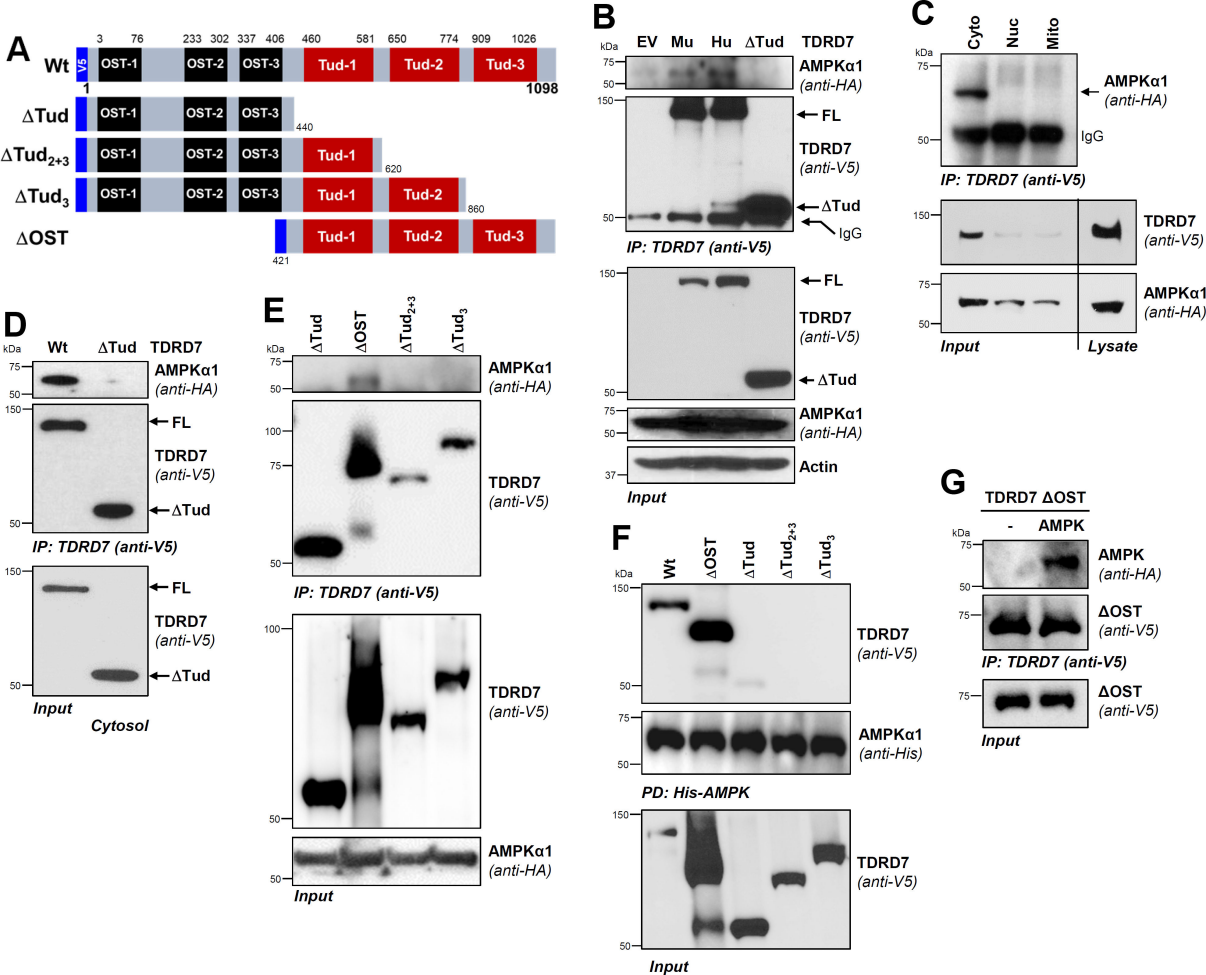
The Tudor-3 domain of TDRD7 is required for interaction with AMPK. (**A**) Wild-type (Wt) TDRD7 with its functional domains (OST and Tud) and the deletion mutants used in the study. (**B**) HEK293T cells were co-transfected with V5-TDRD7 (Hu, human; Mu, mouse), V5-Tdrd7 (mouse), or Tud-deleted mutant (ΔTud) and HA-AMPKα1 plasmids. TDRD7 was immunoprecipitated from the cell lysates, and the immunoprecipitates were analyzed for AMPK by immunoblot. (**C**) HEK293T cells were co-transfected with V5-TDRD7 and HA-AMPKα1 plasmids. TDRD7 was immunoprecipitated from cytosolic, mitochondrial, and nuclear fractions, and the immunoprecipitates were analyzed for AMPK by immunoblot. (**D**) HEK293T cells were co-transfected with Wt TDRD7 or the Tudor-deleted mutant (ΔTud) and HA-AMPKα1 plasmids; TDRD7 was immunoprecipitated from cytosolic fractions. The immunoprecipitates were analyzed for AMPK by immunoblot. (E) HEK293T cells were co-transfected with V5-TDRD7 deletion mutants and HA-AMPKα1 plasmids, as indicated. TDRD7 was immunoprecipitated from cell lysates, and the immunoprecipitates were analyzed for AMPK by immunoblot. (**F**) HEK293T cells were co-transfected with Wt V5-TDRD7 or the deletion mutants (as indicated) and His-AMPK plasmids; AMPK was pulled down by Ni-NTA-agarose from the cell lysates and analyzed for TDRD7 by immunoblot. (**G**) TDRD7 ΔOST mutant and AMPK were expressed bacterially; the recombinant proteins were used for *in vitro* interaction followed by co-immunoprecipitation, as indicated. FL, full length. The results are representative of at least three experiments.

Since TDRD7 and AMPK co-expression led them to interact, we mapped the interacting domains between the two proteins. We designed N-terminally epitope-tagged TDRD7 mutants by deleting functional [OST (∆OST) and Tudor (∆Tud)] domains (Fig. 2A). We validated, using co-IP assay, both human and mouse TDRD7 proteins interacted with AMPK (Fig. 2B). Deletion of all three Tudor domains (ΔTud) abolished AMPK binding, indicating that Tudor domains were required for AMPK interaction (Fig. 2B). To determine the subcellular localization of TDRD7:AMPK complex, we isolated cytosolic, nuclear, and mitochondrial fractions from the co-expressed cells. Co-IP results indicate that TDRD7:AMPK complex was localized predominantly in cytosolic but not nuclear or mitochondrial fractions (Fig. 2C). We confirmed the requirement of Tudor domain for interaction by co-IP analysis in cytosolic fractions, in which only the full-length TDRD7, but not its ΔTud mutant, interacted with AMPK (Fig. 2D). To further narrow down the interacting domain, we tested additional deletion mutants of TDRD7 (ΔOST, ΔTud_2+3_, and ΔTud_3_) using co-IP, which revealed that ΔOST mutant interacted with AMPK (Fig. 2E). Deletion of either Tud-3 alone (ΔTud_3_) or Tud-2 and Tud-3 together (ΔTud_2+3_), however, blocked AMPK interaction (Fig. 2E), indicating that Tud-3 was critical for TDRD7:AMPK interaction. To confirm these results, we performed reverse co-IP by pulling down His-AMPK, which showed strong interaction with Wt and ΔOST but not with Tud-deleted mutants (Fig. 2F). Therefore, Tudor domains of TDRD7 were sufficient for AMPK binding. To confirm ΔOST fragment was sufficient for directly binding AMPK, we expressed them bacterially and purified the recombinant proteins (Fig. S1). Recombinant ΔOST and AMPK were used for *in vitro* interaction, which confirmed that ΔOST interacted directly with AMPK (Fig. 2G). These results, together, demonstrated that Tud-3 domain of TDRD7 was critical for direct interaction with AMPK.

**Fig 3:**
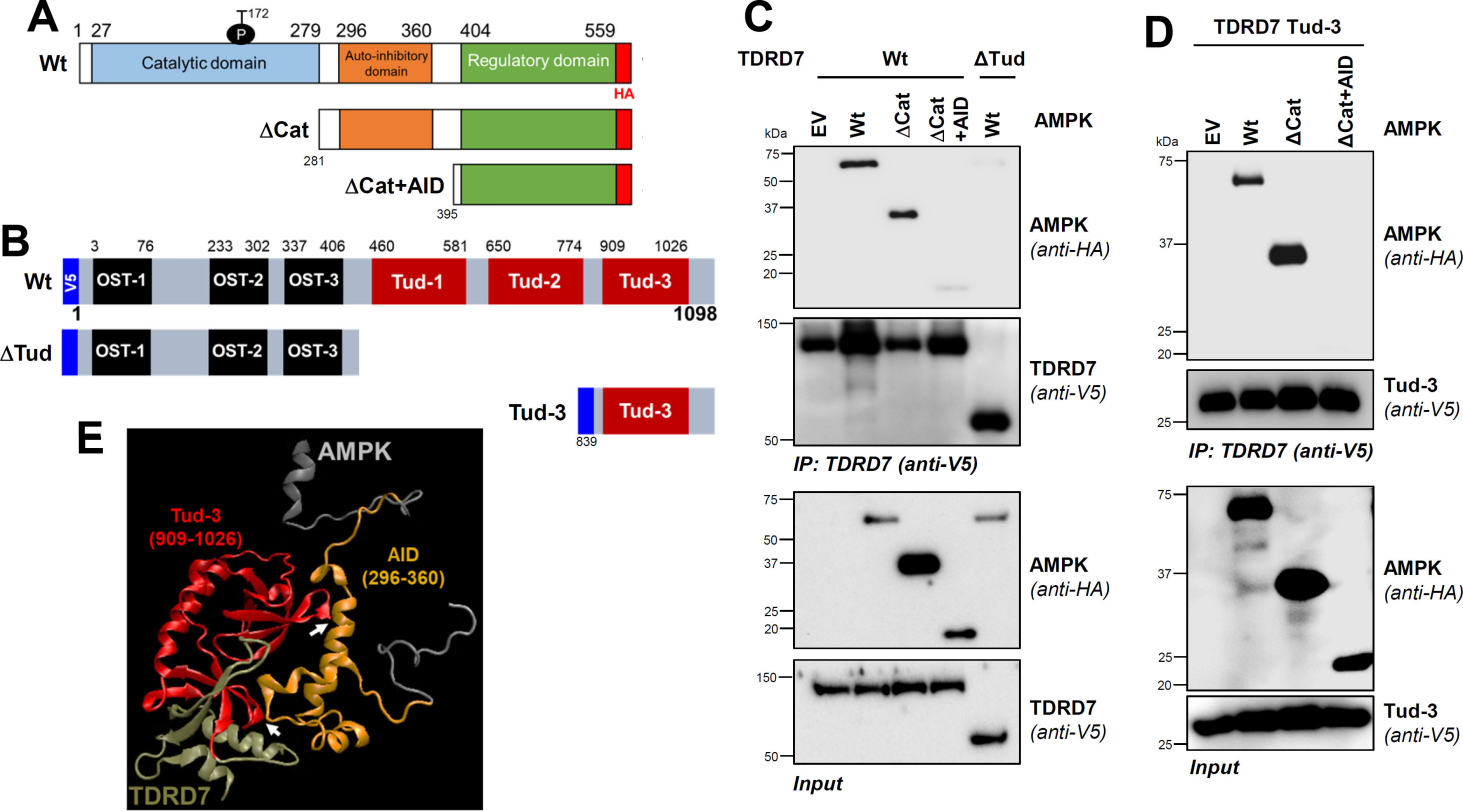
The auto-inhibitory domain (AID) of AMPK is required for interaction with TDRD7. (**A, B**) As shown, wild-type (Wt) and deletion mutants of AMPKα1 and TDRD7 were used for following experiments. (**C**) HEK293T cells were co-transfected with Wt or deletion mutants of HA-AMPK and Wt or Tudor domain-deleted (ΔTud) V5-TDRD7 plasmids, as indicated. TDRD7 was immunoprecipitated from cell lysates, and the immunoprecipitates were analyzed for AMPK by immunoblot. (**D**) HEK293T cells were co-transfected with Wt or deletion mutants of HA-AMPK and the Tud-3 mutant of V5-TDRD7 plasmids, as indicated. TDRD7 was immunoprecipitated from the cell lysates, and the immunoprecipitates were analyzed for AMPK by immunoblot. (**E**) Tudor-3 (Tud-3) and AID domains, as indicated, were used for homology modeling and molecular docking analyses. A modeled structure of the TDRD7:AMPK complex is shown, and arrows indicate the putative contact sites. EV, empty vector. The results are representative of three experiments.

We next mapped TDRD7-interacting domain of AMPK by deleting its critical functional domains, as shown in Fig. 3A. Using co-IP, we revealed Wt and catalytic domain-deleted (ΔCat) AMPK interacted with TDRD7 (Fig. 3B and C). Deletion of auto-inhibitory domain (AID) in ΔCat + AID mutant, however, inhibited TDRD7:AMPK interaction (Fig. 3C). As a negative control, we used ΔTud fragment, which, as expected, did not interact with Wt AMPK (Fig. 3C). These results indicated that AMPK AID was required for interaction with TDRD7. Next, we used C-terminal Tud domain, the Tud-3 fragment (Fig. 3B), and examined its interaction with AMPK and its mutants. Co-IP analyses revealed that Tud-3 interacted with Wt and ΔCat but not with ΔCat + AID, as observed for Wt TDRD7 (Fig. 3D). These results demonstrated that Tud-3 was sufficient for interacting with AID of AMPK. Domain mapping results, together, enabled us to model a putative TDRD7:AMPK complex by molecular docking of the Tud-3 and AID fragments, as we have done previously with other proteins (25, 26). Modeled complex indicated putative contact sites between the two proteins (Fig. 3E). Together, TDRD7 and AMPK interacted directly using specific domains.

### TDRD7:AMPK interaction is required for antiAMPK and antiviral activities of TDRD7

**Fig 4:**
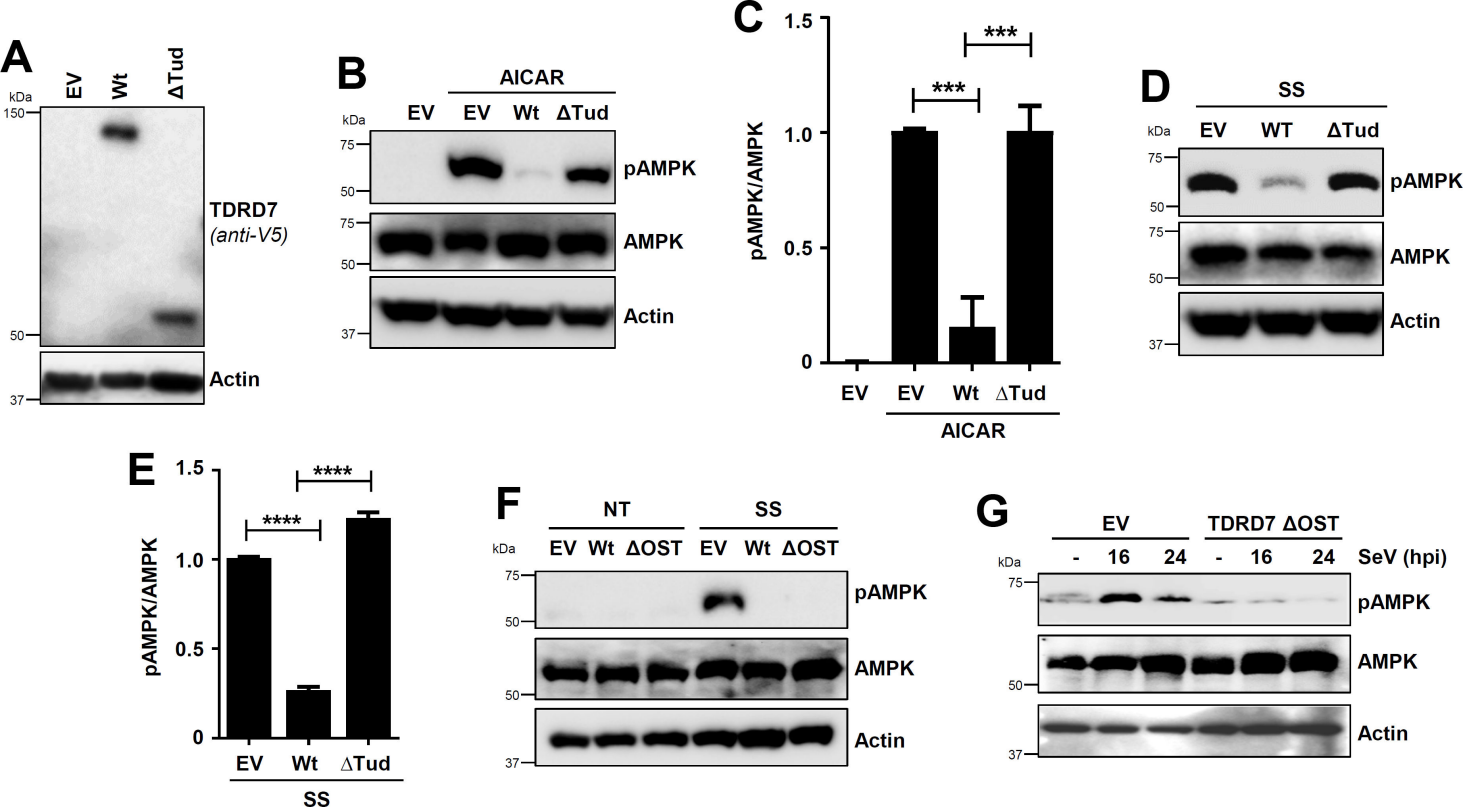
TDRD7:AMPK interaction is required for AMPK inhibition by TDRD7. (**A**) Expression levels of Wt of ΔTud mutant of TDRD7 in TDRD7 KO HT1080 cells were analyzed by immunoblot. (**B, C**) The cells (as in panel A) were treated with AICAR (for 2 h), and pAMPK was analyzed by immunoblot (**B**), which was quantified by Image J (**C**). (**D, E**) The cells (as in panel A) were serum starved (SS, 2 h), and pAMPK was analyzed by immunoblot (**D**), which was quantified by Image J (**E**). (**F**) TDRD7 KO HT1080 cells, stably expressing Wt or ΔOST mutant of TDRD7, were left untreated (NT) or serum starved (SS, 2 h), and pAMPK was analyzed by immunoblot. (**G**) TDRD7 KO HT1080 cells, stably expressing ΔOST mutant of TDRD7, were left untreated (NT) or infected with SeV, and pAMPK was analyzed by immunoblot. The results are representative of three experiments; the data represent mean ± SEM, *P < 0.05.

To determine whether TDRD7:AMPK interaction is responsible for TDRD7-mediated inhibition of AMPK, we expressed Wt TDRD7 (AMPK interacting) and ΔTud mutant (AMPK noninteracting) in TDRD7 KO human cells at similar protein levels (Fig. 4A). In control (empty vector [EV]) cells, AICAR induced robust pAMPK, which was inhibited by Wt TDRD7 but not ΔTud (Fig. 4B and C). SS-induced pAMPK, similarly, was also inhibited by Wt TDRD7 but not ΔTud (Fig. 4D and E). These results indicated that Tudor domains were critical for inhibiting AMPK. Next, we examined whether ΔOST, which interacted directly with AMPK, was sufficient for AMPK inhibition. SS-induced pAMPK, indeed, was strongly inhibited by both Wt and ΔOST mutant of TDRD7, compared to control (EV) cells (Fig. 4F). Similarly, SeV-mediated pAMPK was also inhibited in ΔOST-expressing cells (Fig. 4G). These results demonstrated that ΔOST was sufficient for antiAMPK activity of TDRD7.

**Fig 5:**
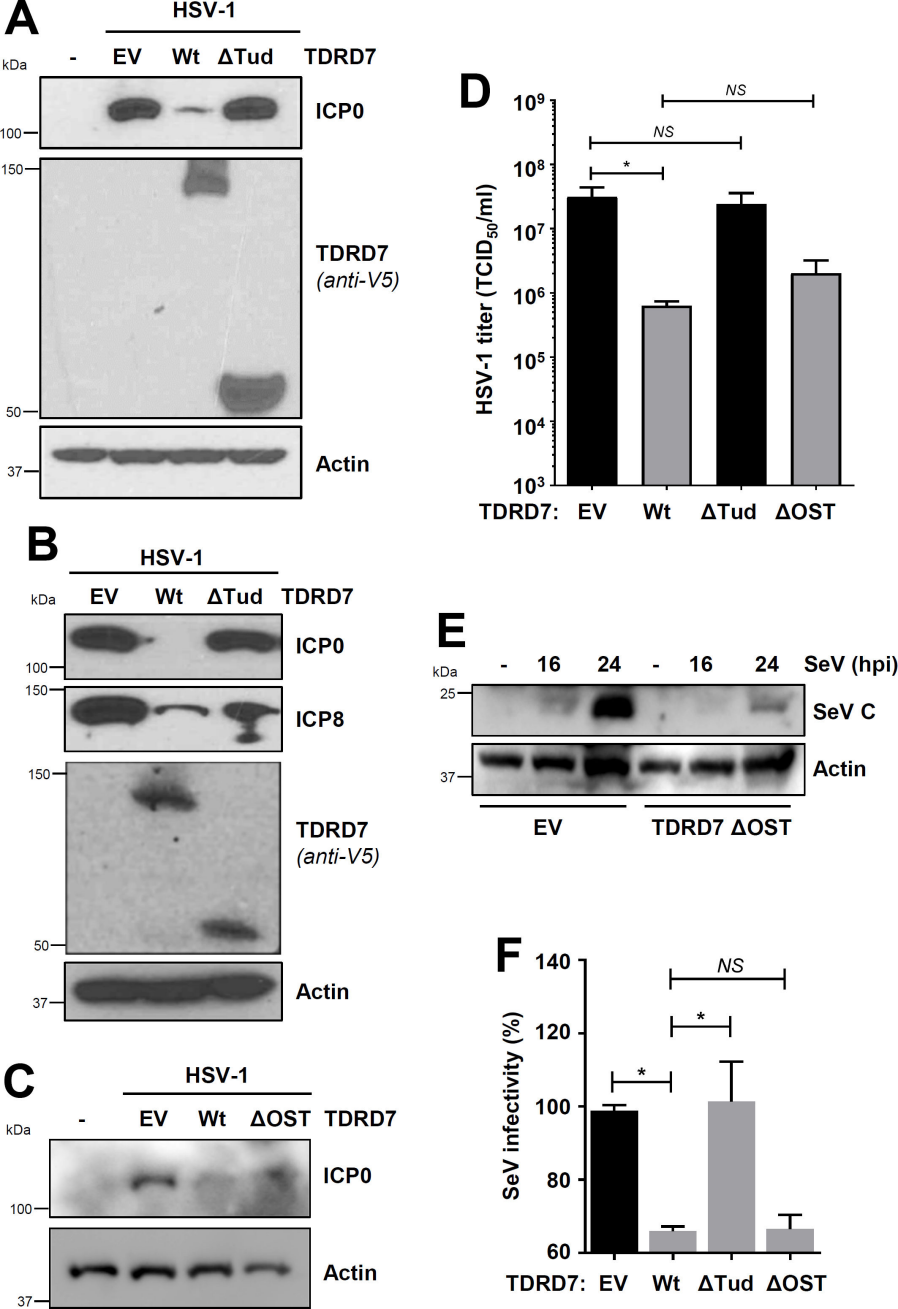
AMPK interaction is required for the antiviral activity of TDRD7 against HSV-1 and SeV. (**A**) HEK293T cells, transfected with Wt or ΔTud mutant of TDRD7, were infected with HSV-1 (multiplicity of infection [MOI], 1), and viral protein (ICP0) expression was analyzed 24 hpi by immunoblot. (**B**) TDRD7 KO HT1080 cells, stably expressing Wt or ΔTud mutant of TDRD7, were infected with HSV-1 (MOI, 1), and viral proteins (ICP0 and ICP8) were analyzed 24 hpi by immunoblot. (**C**) TDRD7 KO cells, stably expressing EV, Wt, or ΔOST mutant of TDRD7, were infected with HSV-1, and viral protein (ICP0) expression was analyzed by immunoblot. (**D**) TDRD7 KO cells, stably expressing Wt, ΔTud, or ΔOST mutants of TDRD7, were infected with HSV-1 (MOI, 1), and infectious viral particles were analyzed 24 hpi by TCID_50_/mL. (**E**) HEK293T cells, stably expressing ΔOST mutant of TDRD7, were infected with SeV, and viral protein (SeV C) expression was analyzed by immunoblot. (**F**) HEK293T cells, transfected with Wt, ΔTud, or ΔOST mutants of TDRD7, were infected with SeV, and viral replication was quantified by flow cytometric analyses of viral antigen expression 16 hpi. The SeV antigen expression of EV was considered 100, and all other values were normalized to this. The results are representative of three experiments; the data represent mean ± SEM, *P < 0.05; EV, empty vector; NS, nonsignificant.

In previous studies, we demonstrated that antiviral function of TDRD7 depends on its antiAMPK activity; AMPK inhibitor alleviates the antiviral activity of TDRD7 (10). To investigate this genetically, we used Wt TDRD7 (AMPK inhibitory) and ΔTud (AMPK noninhibitory) against HSV-1 and SeV, which require AMPK activity for viral replication (9, 10). Ectopic expression of Wt TDRD7 but not ΔTud mutant in HEK293T cells inhibited ICP0 expression in HSV-1-infected cells (Fig. 5A). Encouraged by these results, we examined viral replication in stably expressed cells. Stable expression of Wt TDRD7 but not ΔTud mutant inhibited ICP0 and ICP8 expression in TDRD7 KO cells (Fig. 5B). As expected, ΔOST, which was sufficient for interacting with and inhibiting AMPK, suppressed HSV-1 replication, similar to Wt TDRD7 (Fig. 5C). Infectious virion release, as expected, was inhibited by Wt TDRD7 and ΔOST but not ΔTud (Fig. 5D). We further examined the antiviral function of TDRD7 and its mutants against SeV; ΔOST mutant inhibited SeV viral protein (SeV C) expression (Fig. 5E). Antiviral activities of TDRD7 mutants were further quantified using a flow-cytometry-based assay for viral infectivity, which we used previously (9). Wt TDRD7 significantly inhibited SeV replication compared to EV control (Fig. 5F). As expected, ΔOST but not ΔTud mutant also inhibited SeV replication (Fig. 5F). These results demonstrated TDRD7 interacted with AMPK via its Tudor domain to inhibit AMPK activation and virus replication.

### Tdrd7 knockout primary cells and mice exhibit increased AMPK activation, viral replication, and pathogenesis

**Fig 6:**
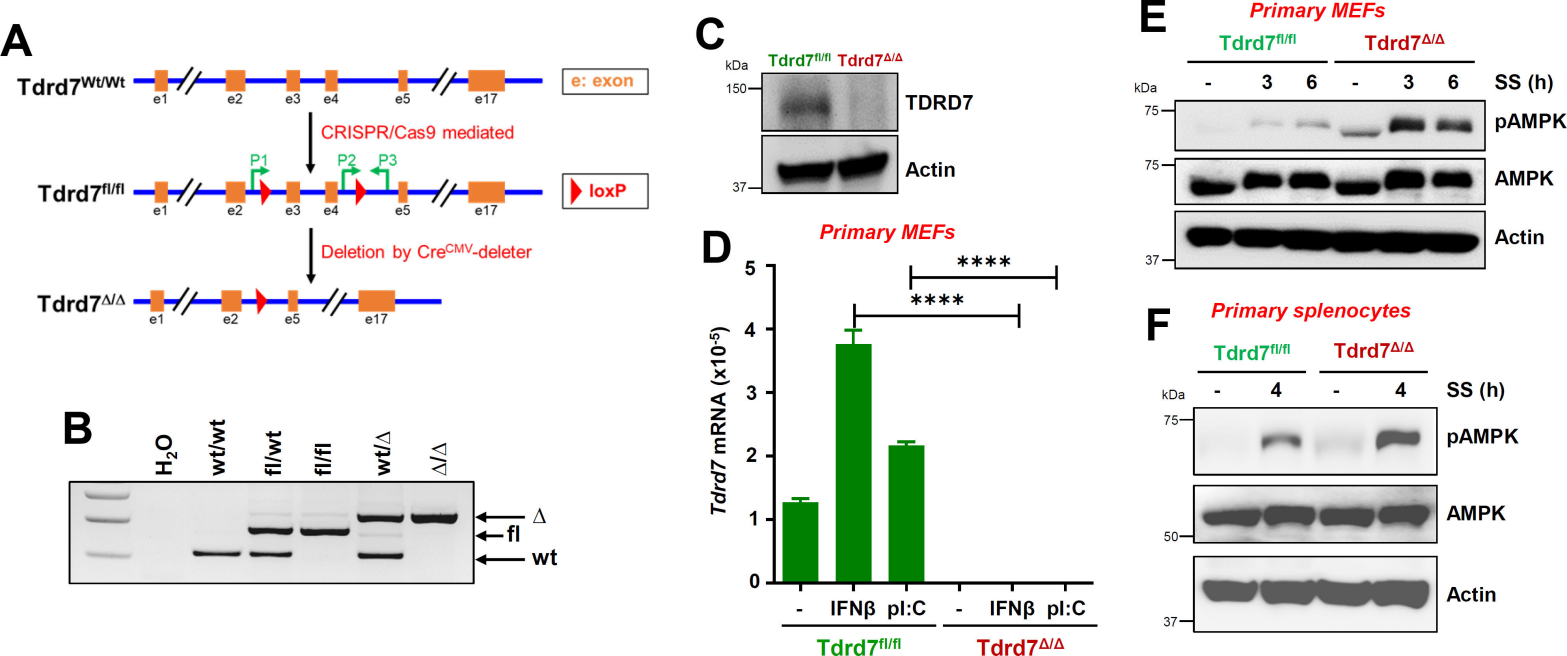
Increased AMPK activation in Tdrd7 knockout primary mouse cells. (**A, B**) A diagram showing the newly generated Tdrd7^fl/fl^ mice and the derived Tdrd7^Δ/Δ^ strain (**A**) and the genotypes of relevant strains (**B**). The location of primers (P) used for genotyping is shown in A. (**C**) Liver homogenates from Tdrd7^fl/fl^ and Tdrd7^Δ/Δ^ mice were analyzed for TDRD7 by immunoblot. (**D**) Tdrd7^fl/fl^ and Tdrd7^Δ/Δ^ primary MEFs were treated with IFNβ or polyI:C for 16 h, and the Tdrd7 mRNA levels were analyzed by quantitative reverse transcription-polymerase chain reaction (qRT-PCR). (**E**) Tdrd7^fl/fl^ and Tdrd7^Δ/Δ^ primary MEFs were serum starved (SS) for the indicated times, and pAMPK was analyzed by immunoblot. (**F**) Tdrd7^fl/fl^ and Tdrd7^Δ/Δ^ primary splenocytes were serum starved (SS) and analyzed for pAMPK by immunoblot. The results are representative of three experiments using two to four mice from each group; the data represent mean ± SEM; *P < 0.05.

To evaluate the relative contribution of Tdrd7 to prevent viral infection and pathogenesis, we generated Tdrd7 conditional knockout mice by crossing floxed (fl)-Tdrd7 (Tdrd7^fl/fl^) mice with Cre^CMV^ deleter mice (Fig. 6A and B). We confirmed Tdrd7 deletion in tissues (Fig. 6C) and primary mouse embryonic fibroblasts (MEFs) (Fig. 6D), isolated from Tdrd7^fl/fl^ and Tdrd7^Δ/Δ^ mice. In primary MEFs, Tdrd7 was expressed in naïve cells, and treatment with IFNβ or transfection of polyI:C strongly upregulated its expression in Tdrd7^fl/fl^ but not in Tdrd7^Δ/Δ^ cells (Fig. 6D). We tested whether endogenous TDRD7 and AMPK proteins interact in mouse tissues; co-IP results from liver homogenates indicated interaction between these proteins (Fig. S2). We examined whether Tdrd7 deficiency led to increased AMPK activation. In primary MEFs, SS-induced pAMPK was enhanced in Tdrd7^Δ/Δ^ MEFs, compared to Tdrd7^fl/fl^ cells (Fig. 6E). In Tdrd7^Δ/Δ^ primary splenocytes, SS-induced pAMPK was increased compared to Tdrd7^fl/fl^ cells (Fig. 6F). Tdrd7 knockout primary cells displayed increased AMPK activation compared to the control cells.

**Fig 7:**
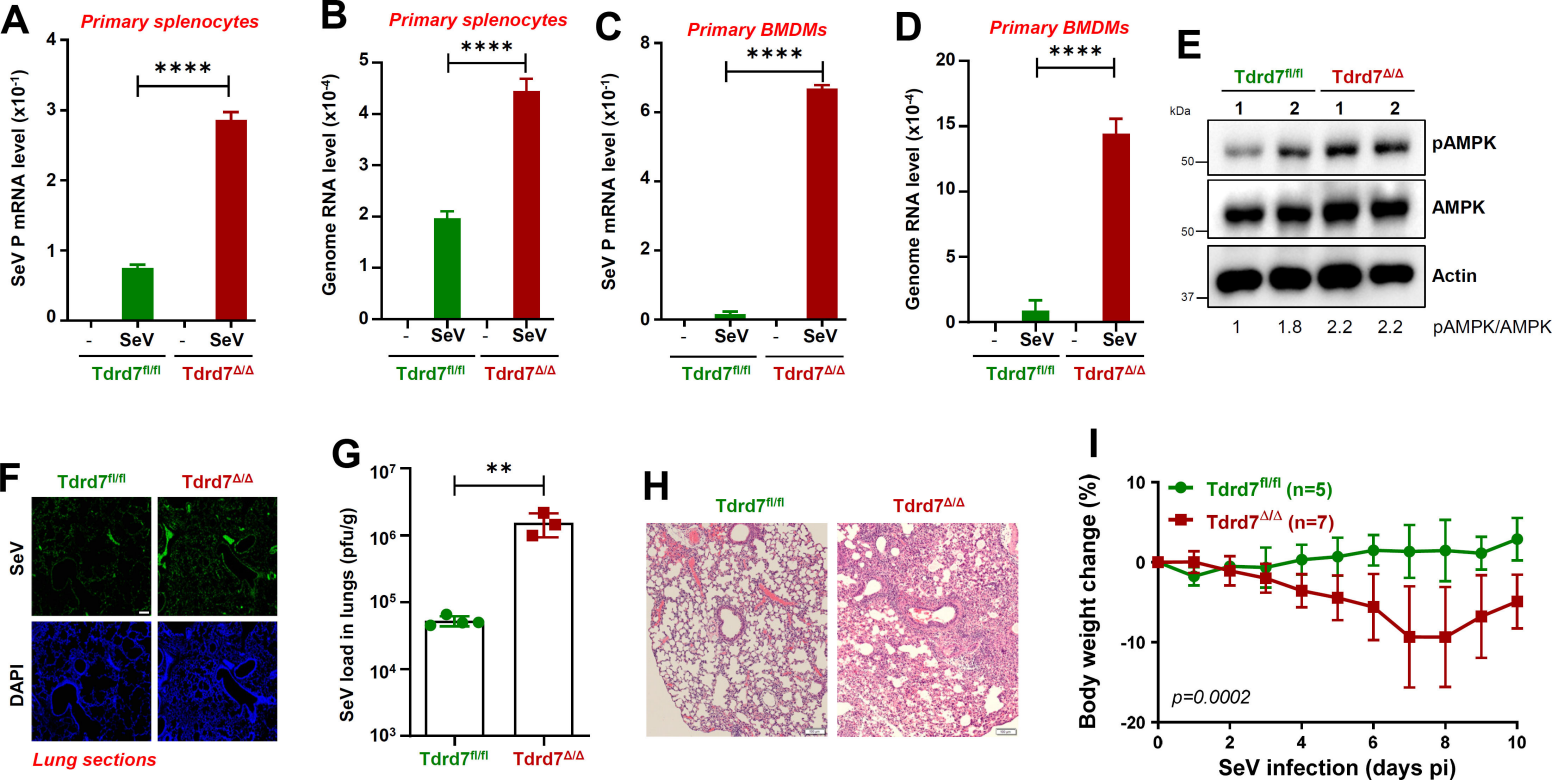
Tdrd7 knockout cells and mice exhibit increased viral load and susceptibility to SeV infection compared to the control. (**A, B**) Tdrd7^fl/fl^ and Tdrd7^Δ/Δ^ primary splenocytes were infected with SeV (MOI, 5; 16 hpi); viral mRNA (SeV *P* gene) and genome levels were analyzed by qRT-PCR. (**C, D**) Tdrd7^fl/fl^ and Tdrd7^Δ/Δ^ primary bone marrow-derived macrophages (BMDMs) were infected with SeV (MOI, 5; 16 hpi); viral mRNA (SeV *P* gene) and genome levels were analyzed by qRT-PCR. (**E**) Tdrd7^fl/fl^ and Tdrd7^Δ/Δ^ mice were infected intranasally with SeV (125,000 pfu/mouse). Lung lysates from two mice from each genotype were analyzed for pAMPK 7 days post-infection (dpi) by immunoblot (**E**). (**F-I**) Tdrd7^fl/fl^ and Tdrd7^Δ/Δ^ mice were infected intranasally with SeV (as in panel E), and the following analyses were performed: lung sections from the SeV-infected mice (7 dpi) were immuno-stained with antiSeV antibody and analyzed by confocal microscopy (F; scale bar, 500 µm); SeV infectious load in lung homogenates was analyzed by plaque assay (**G**); lung sections were H&E stained for histological analyses (H; scale bar, 100 µm), and the infected mice were monitored for body weight post-infection (**I**). The results are representative of three experiments; the data represent mean ± SEM, *P < 0.05.

We next examined viral load in Tdrd7^Δ/Δ^ primary cells; in Tdrd7^Δ/Δ^ primary splenocytes and BMDMs, levels of SeV transcript and genomic RNA were enhanced, compared to Tdrd7^fl/fl^ cells (Fig. 7A through D). To investigate the role of Tdrd7 against viral pathogenesis, we infected Tdrd7^fl/fl^ and Tdrd7^Δ/Δ^ mice intranasally with SeV and analyzed for pAMPK and viral antigen levels in infected lungs and monitored body weight changes. Tdrd7^Δ/Δ^ mouse lungs showed increased pAMPK compared to control mice (Fig. 7E). Tdrd7^Δ/Δ^ mouse lungs showed increased SeV antigen levels, compared to control mice, as analyzed by immunohistochemistry (Fig. 7F; S3A). Infectious viral load also significantly enhanced in Tdrd7^Δ/Δ^ mouse lungs compared to the control mice (Fig. 7G). Elevated levels of viral antigen expression and infectious viral load in Tdrd7^Δ/Δ^ mice led to increased lung pathology. hematoxylin and eosin (H&E) staining revealed increased immune cell infiltration in Tdrd7^Δ/Δ^ lungs, compared to Tdrd7^fl/fl^ control (Fig. 7H; S3B). As a result, Tdrd7^Δ/Δ^ mice were more susceptible to infection, as analyzed by loss in body weight, compared to the Tdrd7^fl/fl^ mice (Fig. 7I). In summary, Tdrd7 deficiency led to increased AMPK activation and viral infection in primary cells and mice.

## DISCUSSION

**Fig 8:**
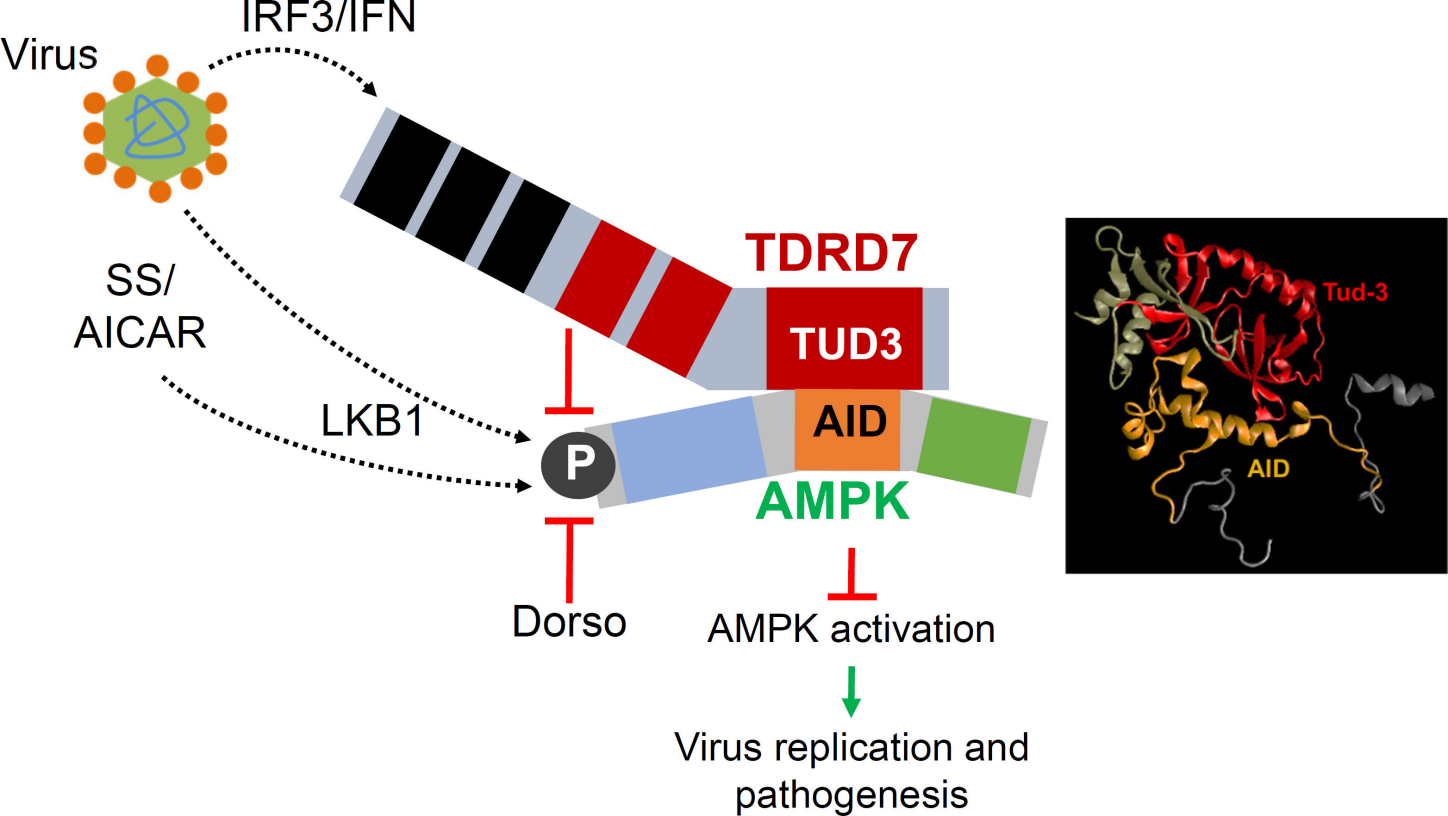
A model representing IFN-inducible protein TDRD7 interacts with pro-viral AMPK and inhibits its activation to suppress viral replication and pathogenesis. Virus infection, via IRF3/IFN pathway, transcriptionally induces TDRD7, which interacts with AMPK via the C-terminal Tudor domain, to inhibit AMPK activation. Various inducers, e.g., virus infection, serum starvation (SS), or AICAR, activate LKB1, the kinase that directly phosphorylates AMPK. Inhibition of AMPK activation by TDRD7 is a molecular basis for its viral restriction activity. The TDRD7-AMPK branch is a novel antiviral pathway of the IFN system.

In previous studies, we showed TDRD7 is a novel ISG and inhibits virus-induced autophagy by interfering with AMPK activation to suppress virus replication. Here, we demonstrated a molecular mechanism for TDRD7-mediated inhibition of AMPK (Fig. 8). TDRD7 interacted directly with AMPK via specific domains, and the interaction was required for its antiAMPK functions. TDRD7 consists of RNA-binding domains (OST), and arginine-methylated protein binding domains (Tudor) (27). Our results suggest that C-terminal Tudor domain (Tud-3) of TDRD7 interacted directly with AID of AMPK. Molecular modeling of TDRD7:AMPK complex revealed a putative interface between Tud-3 and AID, and future studies will further narrow down the specific amino acids involved in this interaction. Since AMPK gets activated by LKB1:AMPK complex formation on lysosome membrane, we speculate that TDRD7 likely targets activation step by disrupting the complex. AMPK activators are used clinically to treat diabetes (28, 29); however, unregulated AMPK activities may be harmful. A small peptide of TDRD7 blocking AMPK may inhibit these effects. The TDRD7:AMPK interacting domains may also be therapeutically targeted for potential anti-AMPK and antiviral compounds. Viruses, however, can evade cellular antiviral response by inhibiting this complex. Autophagy-regulatory activity of TDRD7 has also been demonstrated in lens development and spermatogenesis by preventing autophagosome from fusion with lysosome (30). Therefore, TDRD7 likely regulates autophagy by targeting multiple steps of the pathway.

Using Tdrd7 knockout mice and derived primary cells, we evaluated the physiological relevance of Tdrd7 functions. Tdrd7 knockout primary cells exhibited enhanced AMPK activation and elevated viral load. Tdrd7 knockout mice displayed increased susceptibility to SeV pathogenesis compared to the Wt control. A recent study using transcriptomic screens of humans, ferrets, and mice revealed TDRD7 as an antiviral ISG against influenza A virus (31). TDRD7 is known to localize in cytoplasmic RNA granules to regulate lens development (32). TDRD7 regulates the mRNAs critical for lens development, and as a result, Tdrd7 mutant mice develop cataracts. A recent study showed that TDRD3 is an antiviral protein and functions via recruitment to stress granules (SGs) and modulating innate immune signaling (33). TDRD3 containing SGs recruit effector innate immune signaling proteins, TRAF3 and IRF7. Whether TDRD7 also functions via SGs for its antiviral response will require future investigation. The autophagy-regulating functions of TDRD7 may also be related to its SG localization and the ability to bind RNA.

Our results have implications for interplay between IFN response and metabolic pathway. IFN has earlier been shown to trigger intracellular PI3K signaling to interfere with AMPK activation (34). Therefore, TDRD7 may be an effector ISG for IFN-mediated inhibition of AMPK, suggesting that an IFN-inducible protein can further amplify IFN’s anti-AMPK effects. Because viruses utilize AMPK differentially, it will be interesting to investigate whether IFN-mediated direct AMPK inhibition has any role in viral infection, particularly where IFN is deleterious to the host. On the other hand, AMPK has been shown to regulate IFN synthesis and signaling (22, 23). AMPK regulation by an ISG may provide a regulatory circuit for cellular IFN response. Because AMPK has largely been studied in the context of autophagy, its role in autophagy-independent response is unclear. Our recent study in autophagy-deficient cells revealed that HSV-1 utilizes an autophagy-independent function of AMPK (10, 35). Future studies will reveal the nature of such a function and whether IFN and its induced proteins may also influence those. Since IFN treatment or virus infection triggers induction of many ISGs, some may also share anti-AMPK function. Screening of an AMPK-interacting motif may help identify additional anti-AMPK ISGs.

How viruses activate and utilize AMPK is unclear; the answer depends on the virus (16). Our studies indicate that SeV takes advantage of the AMPK-autophagy pathway for effective replication, whereas HSV-1 utilizes an autophagy-independent function of AMPK (9). AMPK is a well-studied metabolic kinase, and its activation is regulated by cellular energy levels (12, 13). Low cellular AMP causes activation of LKB1, which directly phosphorylates Thr^172^ of AMPK. Active AMPK is a protein complex of α, β, and γ subunits, which form a productive assembly for subsequent downstream functions. AMPK α subunit has α1 and α2 isoforms, which expressed cell type specifically and activated simultaneously via phosphorylation of consensus Thr^172^ residue (13, 36). In addition to LKB1, AMPK can be directly phosphorylated by CaMKK, which gets activated by increased Ca^2+^ levels. Many viruses cause increased Ca^2+^ levels during infection, activating AMPK via CaMKK. In the current study, we focused on the phosphorylation of Thr^172^, which leads to downstream functions of AMPK. Moreover, AMPK activation is regulated by other posttranslational modifications, e.g., AKT-mediated phosphorylation of Ser^485^ inhibits phosphorylation of Thr^172^ (21, 36 – 38). Hepatitis C virus regulates AMPK activity using this mechanism (21). Alternatively, TDRD7 may interact with AMPK to recruit AKT to trigger phosphorylation of Ser^485^. Future studies will test a possibility that TDRD7 triggers dephosphorylation of pThr^172^ by activating and recruiting the phosphatase PP2A (39). However, TDRD7 may rely on these strategies to efficiently inhibit the proviral AMPK activity.

In summary, our results provide a molecular mechanism for IFN-mediated inhibition of AMPK. The mechanism may apply to other protein inhibitors of AMPK and other ISGs that exhibit antiviral activity by inhibiting AMPK. TDRD7 mutations lead to cataract formation, and studies are required using the cells from these individuals to determine whether AMPK pathways are altered and whether AMPK activity regulates lens development. Primary cells from individuals with TDRD7 mutations would further provide a translational potential of our results.

## MATERIALS AND METHODS

### Cells, plasmids, and reagents

Human cell lines HEK293T, HT1080, and HeLa were purchased from ATCC (Manassas, VA, USA); TDRD7 KO HT1080 cells and L929 cells expressing Tdrd7-specific shRNA were described previously (9). All cell lines used in this study were maintained in the authors’ laboratory. The cells were maintained in Dulbecco's modified Eagle medium (DMEM) containing 10% fetal bovine serum, penicillin, and streptomycin. AMPK activator (AICAR) was obtained from Selleckchem, and Lipofectamine 2000 was obtained from Thermo Fisher Scientific. The antibodies against the specific proteins were obtained as indicated below: anti-SeV C and anti-whole SeV antibodies were described before (9), anti-TDRD7 (Sigma-Aldrich #SAB1303547), anti-LC3 (Cell Signaling #2775), anti-pAMPK (Cell Signaling #2535), anti-AMPK (Cell Signaling #2532, Thermo Fischer Scientific #MA5-15815), antiactin (Sigma-Aldrich #A5441), anti-V5 (Thermo Fisher Scientific #R960-25 and Cell Signaling #13202S), anti-HA (Abcam #ab18181 and CST #3724), anti-His (Qiagen #34660), anti-Flag (CST #2368S), anti-ICP0 (Abcam #ab6513 and Santa Cruz #sc-53070), anti-ICP8 (Santa Cruz #53329), and the goat anti-mouse and goat anti-rabbit secondary antibodies were obtained from Rockland.

### Knockdown and ectopic expression

For generating stable Tdrd7 knockdown mouse cells, the shRNA CAGGATTTGCCTCA GATTA was lentivirally expressed, and transduced cells were selected in the puromycin-containing medium, as described before (9). The stable knockdown cells were evaluated for levels of Tdrd7 by qRT-PCR. Human cell lines, stably expressing epitope (V5) tagged Wt or mutants of TDRD7, were generated in TDRD7 KO HT1080 cells. These cells were generated by lentiviral transduction followed by selection in the puromycin-containing medium. The stable cells were used for viral infection and other biochemical analyses.

### Virus infections

SeV Cantell (VR-907) and SeV 52 (VR-105) strains (Charles River) and HSV-1 KOS strain (40), and the infection procedures have been previously described (10). Briefly, the cells were infected by the viruses (at an multiplicity of infection [MOI] specified in the figure legends) in serum-free DMEM for 2 h, after which the cells were washed and replaced with the normal growth medium. The virus-infected cells were analyzed at indicated time for viral protein expression or as described in figure legends. For quantification of infectious HSV-1 particles in the culture medium, the TCID_50_ assay was performed as described previously (10, 41).

### Treatment of cells for analysis of AMPK activation

For analyses of AMPK activation through serum starvation, the cells were washed with and incubated in serum-free DMEM for 4 h or the time period indicated in figure legends or treated with 500 µM of AICAR for 2 h. The cells were harvested, and pAMPK was analyzed by immunoblot.

### Cell lysis and immunoblot

Immunoblot analyses were performed using previously described procedures (9, 42). Briefly, cells were lysed in 50 mM Tris buffer, pH 7.4 containing 150 mM of NaCl, 0.1% Triton X-100, 1 mM sodium orthovanadate, 10 mM of sodium fluoride, 10 mM of β-glycerophosphate, 5 mM sodium pyrophosphate, protease, and phosphatase inhibitors (Roche). Total protein extracts were analyzed by SDS-PAGE followed by immunoblot. The immunoblots were developed using a Syngene imaging system and processed using Adobe Photoshop for further analyses. The density of protein bands on the immunoblots was quantified, wherever indicated, using the Image J program.

### Generation and expression of TDRD7 and AMPK mutants

Expression vectors of human and mouse, TDRD7/Tdrd7 gene, were obtained from Origene and subcloned with V5-tag into lentiviral vector pLVX-IRES-puro. AMPK expression plasmid was obtained from Addgene and was subcloned into pLVX-IRES-puro as HA or His-tagged AMPK. The TDRD7 and AMPK mutants were generated either by megaprimer PCR or NEB site-directed mutagenesis kit, and the mutations were confirmed by sequencing and immunoblot analysis. Recombinant TDRD7 (ΔOST, doubly-tagged with His and V5) and AMPK (doubly-tagged with His and HA) proteins were purified from *Escherichia coli* BL-21 strain using the previously described procedure (43).

### Co-immunoprecipitation and *in vitro* protein-protein interaction assay

Co-immunoprecipitation assays were performed by co-transfection of epitope (HA or His)-tagged AMPK and V5-tagged TDRD7 constructs for 24 h followed by lysis in 3-[4-(2-hydroxyethyl)piperazin-1-yl]propane-1-sulfonic acid (EPPS) buffer containing protease inhibitors by repetitive freeze-thaw cycles, and lysates were immunoprecipitated with anti-V5-agarose beads or pulled down with Ni-NTA-agarose beads overnight at 4°C. Beads were washed twice with EPPS buffer and once with RIPA buffer, and bound proteins and eluates were analyzed by immunoblot. For *in vitro* interaction, bacterially purified TDRD7 and AMPK proteins were incubated at 37°C for 2 h, and co-IP assays were performed. For co-IP of endogenous TDRD7 and AMPK proteins, mouse liver homogenates were prepared in lysis buffer and immunoprecipitated by anti-AMPK antibody followed by immunoblot with anti-TDRD7 antibody.

### Molecular docking analyses

The AMPK three-dimensional structure was modeled on template 6C9J, and the TDRD7 structure was built on template 5m9n.2 based on sequence homology using SWISS-MODEL because there was no structure available for full-length TDRD7. The templates were selected based on the close sequence similarity and were used for docking analysis. The 3D model structure of the AMPK:TDRD7 complex was predicted using Z-DOCK. The auto-inhibitory domain of AMPK and Tudor domain of TDRD7 were used for binding, and non-interacting residues were blocked in the input for predictions from Z-DOCK. The top 10 predictions were analyzed, and the most likely conformation was selected based on the binding affinity and optimal binding arrangement as a representative.

### Isolation of subcellular fractions

Cytosolic, nuclear, and mitochondrial fractions from transfected cells were isolated as described previously using the Mitochondria Isolation Kit (Pierce Biotechnology), following the manufacturer’s instructions (44). Isolated mitochondrial and nuclear fractions were washed with phosphate-buffered saline (PBS) and extracted in lysis buffer for co-immunoprecipitation and immunoblot analysis.

### RNA isolation and qRT-PCR analyses

Total RNA was isolated using Trizol extraction, and cDNA was prepared using ImProm-II Reverse Transcription Kit (Promega). For qRT-PCR, 0.5 ng of cDNA was analyzed using Radiant SYBR Green reagent (Alkali Scientific) on Roche Light Cycler. Expression levels of the mRNAs were normalized to 18S rRNA. For the qRT-PCR analyses of the respective genes, the following primers were used: Tdrd7-fwd, CTAAGGGCTGTCCTGCAGTC; Tdrd7-rev, AGAGTTGCCTTTGGCTTT; SeV P-fwd, CAAAAGTGAGGGCGAAGGAGAA; SeV P-rev, CGCCCAGATCCTGAGATACAGA; SeV genome-fwd, ACTGGTCCGGATAAGAAGGC; SeV genome-rev, AGTTCCTGATCAGACCCGTG; 18S-fwd, ATTGACGGAAGGGCACCACCAG; 18S-rev, CAAATCGCTCCACCAACTAAGAACG.

### Flow cytometry and analysis

HEK293T cells were transfected with TDRD7 Wt or mutants; 24 h later, the cells were infected with SeV (Cantell) at an MOI of 10. After 16 h, cells were harvested, fixed with 1% paraformaldehyde in PBS for 10 min, permeabilized with 0.1% (wt/vol) Triton X-100, and incubated with an anti-SeV polyclonal antibody and an Alexa Fluor conjugated secondary antibody. Cells were analyzed on BD Canto (flow cytometer) using Flow Jo. The relative infectivity in each condition was normalized to EV to obtain percent infection.

### Confocal microscopy and proximal ligation assay

HEK293T cells were grown on coverslips and transfected with HA-AMPK and V5-TDRD7. Twenty-four hours post-transfection, the cells were fixed in 4% paraformaldehyde and permeabilized in 0.2% Triton X-100 and immunostaining by anti-HA and anti-V5 antibodies followed by Alexa Fluor-conjugated secondary antibody. Objects were mounted on slides using VectaShield/DAPI and analyzed by confocal microscopy. For studying interaction of endogenous proteins, HT1080 cells were grown on coverslips and immuno-stained with anti-TDRD7 and anti-AMPK antibodies, followed by Alexa Fluor-conjugated secondary antibodies. Images were further processed and analyzed using Adobe Photoshop software. Multiple culture fields were analyzed to select representative images. For the proximal ligation assay, the immunostained (with anti-HA and anti-V5 antibodies) cells were used for duolink assay (DUO92008-3, DUO92004, DUO92002, Sigma-Aldrich) using manufacturer’s instructions and as described previously (26).

### Mice, primary cells, and virus infection

The Tdrd7^fl/fl^ mice were custom generated by Cyagen Inc, using the design of the target allele indicated in Fig. 6A. The Tdrd7^fl/fl^ mice were crossed with Cre^CMV^ mice (from JAX) to obtain the Tdrd7^Δ/Δ^ mice and used for our studies. The genotyping of the mice was performed using the following primers: P1, GTGCTGAATGCCTGAAAGATGATG; P2, CTCATTGTTTTATGTTGTGCGGAC; and P3, CAACACAGGCCATACATGAACAGAG (as shown in Fig. 6A). For virus infection studies, the mice were infected with SeV 52 strain intranasally and were monitored every day for changes in body weight. The lungs were harvested 7 dpi, when the total RNA was isolated and analyzed by qRT-PCR. For isolation of primary cells (e.g., MEFs, splenocytes, and BMDMs), previously described procedures were used (42). Infectious SeV load in the lungs was evaluated by previously described method (42). Briefly, the infected lungs were weighed and homogenized in PBS, and viral titrations were carried out using LLCMK2 cells. All animal procedures are approved by the University of Toledo Institutional Animal Care Committee (IACUC).

### Immunofluorescence staining

Lungs from SeV-infected mice were harvested and fixed in 10% neutral buffered formalin. These were then sliced at 5 µm followed by paraffin embedding. Before immunostaining, the lung sections were deparaffinized, antigen retrieved, and blocked using 10% horse serum for an hour before overnight incubation with primary antibody against SeV antigen (9). Sections were washed with Tris-buffered saline with Tween-20 (TBST) and incubated with Alexa Fluor 488 conjugated anti-rabbit (A32731, Invitrogen). The sections were washed and mounted on the glass slides using VectaShield/DAPI mounting media (H-1200, Vector Laboratories). The stained sections were imaged using confocal microscopy and analyzed using OlyVia (OLYMPUS OlyVIA 2.9). H&E staining for the lung sections was performed as described previously (26, 43).

### Statistical analyses

Statistical analyses were performed using GraphPad Prism 9 software or Microsoft Excel for Windows 10. The *P* values were calculated using two-tailed, unpaired Student’s *t*-tests (for comparing two groups) or one-way analysis of variance (ANOVA) (for comparing more than two groups), based on the number of sets for comparison. *P* < 0.05 was considered statistically significant. The results presented here are representatives of three or more independent experiments.

## SUPPLEMENTAL MATERIAL

Supplemental figures mbio.00611-23-s0001.pdf Figures S1 to S3.

ASM does not own the copyrights to Supplemental Material that may be linked to, or accessed through, an article. The authors have granted ASM a non-exclusive, world-wide license to publish the Supplemental Material files. Please contact the corresponding author directly for reuse.
