# Supplementary material for: Interferon-stimulated gene TDRD7 interacts with AMPK and inhibits its activation to suppress viral replication and pathogenesis
Source: mBio. 2023 Sep 15;14(5):e00611-23. doi: 10.1128/mbio.00611-23 (PMC10653931; doi:10.1128/mbio.00611-23)
Supplement: Supplemental figures — Figures S1 to S3. [file mbio.00611-23-s0001.pdf]

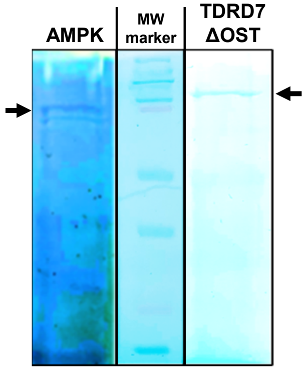

Fig S1. Recombinant TDRD7 and AMPK proteins for *in vitro* binding. TDRD7 ΔOST mutant and full-length AMPK were expressed in *E. coli* and the recombinant proteins were purified and Coomassie stained. MW, molecular weight.

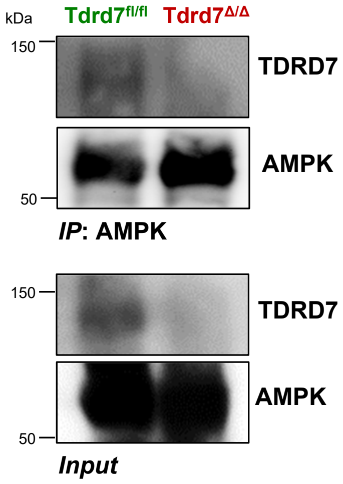

Fig S2. Endogenous TDRD7 and AMPK proteins interact in liver tissues. Liver homogenates from Tdrd7$^{fl/fl}$ and Tdrd7$^{\Delta/\Delta}$ mice were used for co-immunoprecipitation analyses, as indicated.

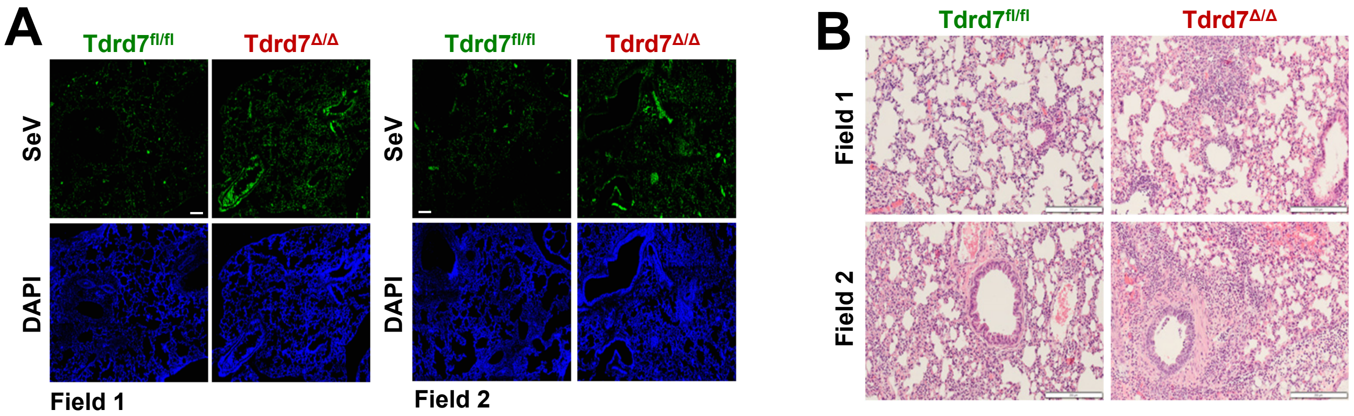

Fig S3. Tdrd7 knockout mice are more susceptible to SeV infection compared to the control mice. Tdrd7$^{fl/fl}$ and Tdrd7$^{\Delta/\Delta}$ mice were infected intranasally with SeV (125,000 pfu/mouse). Lung sections from the SeV-infected mice (7 dpi) were immuno-stained with anti-SeV antibody and analyzed by confocal microscopy (A, Scale bar: 500µm), H&E-stained for histological analyses (B, scale bar: 200 µm). Two different fields are shown for A and B.
